# Dentine Plastique Du Docteur Klein

**Published:** 1900-02

**Authors:** I. B. Davenport


### Domestic Correspondence.

### DENTINE PLASTIQUE DU DOCTEUR KLEIN.

BY I. B. DAVENPORT, M.D.

To the Editor:

Sir,—The following paper read before the American Dental Club of Paris, in October, 1899, with the accompanying protest, was voted by the Club, November 4, 1899, to be sent for publication to leading dental journals of Europe and America.

Mr. President and Gentlemen,—During the winter of 1897–98, a man calling himself Dr. Klein, of Buda-Pesth, came to Paris to sell his interest in a pulp-capping which he called "Dentine Plastique du Dr. Klein."

He sent around samples to dentists for trial, and a few weeks later called to ask for a testimonial.

During that time he sent to the club, for our examination, a section of tooth, filled with cement, over a capping which rested upon the bottom of the pulp-chamber; this was accompanied by the statement: "That this capping and filling had been done in the mouth three years before the tooth had been extracted; the extraction having been done for the scientific purpose of showing the transformation, during the three years' sojourn in the mouth, of the capping material into organized dentine."

We all examined the section under the microscope, and easily remember the peculiar appearance of the capping.

Soon after, a call from this same gentleman on several members of the club was made, to request a testimonial, which was refused; but not long after we all received a circular (which I here brought along) recounting the virtues of the plastic-capping, and containing an engraving of the sections of tooth sent to us for inspection, with the names of nearly all of us appended to a testimonial in favor of this material.

Nearly all the names on that circular were placed there either directly against a specific refusal to allow their use, or without being asked.

This circular appears to have been published all over the world (it lately appeared in a Mexican dental journal) with our names added against our wish or knowledge, and is helping to sell this stuff of whose composition we know nothing.

Now, about that piece of tooth sent for us to inspect, and this drawing made from the same, which forms part of the circular to which our names are appended, Mr. Dalton can tell you, and has kindly consented to do so.

Mr. Dalton is an expert preparer of objects for microscopical study; his work in the form of "Dalton's Gems" is well known.

*Mr. Dalton.*—Some time ago a friend brought me a section of a tooth, of which a gentleman, unknown to me, wished a drawing made, showing particularly the appearance of a substance under the filling. This cut in the circular mentioned by the essayist is a reproduction of the drawing made by me, excepting that these heavy lines have been made over my drawing, as if marking it off into little squares; in all other particulars it is as I drew it.

When I examined the section from which the drawing is made, I noticed a familiar substance occupying part of the pulp-chamber and supporting the filling. I scratched out a slight fragment, placed it under the microscope, and found it to be as I first supposed, a section of cuttle-fish bone.

I am not a dentist, but when my friend came for the drawing, I casually remarked, "That must have been a clever idea to place a light support of cuttle-fish bone to build the filling upon." My friend replied, "Get out with your cuttle-fish bone! that is new-formed dentine."

I was greatly surprised, as I supposed he knew its composition. After some difficulty he was convinced, but not until I had shown him, under the microscope, pieces of this capping side by side with pieces cut out of cuttle-fish.

Gentlemen, from Mr. Dalton's remarks, you see the imposition shown in that section of tooth; and in this drawing, which has marked over it in large letters:[1]

It seems evident that this device was intended solely to make a favorable impression, and facilitate the sale of a product which, if as good as pretended, ought not to need so much misrepresentation.

This improper use of our names gives a false impression.

Mr. Dalton then showed under the microscope preparations of cuttle-fish bone which were made on the spot.

PROTEST.

We, the undersigned, whose names are appended to the affirmation of the success of "Dentine Plastique du Dr. Klein," have never given consent to the use of our names in that connection.

| | |
|---|---|
| J. G. BRIGIOTTI, | JOHN EVANS, |
| E. A. BOGUE, | J. MICHAELS, |
| G. C. DABOLL, | A. HUGUENSCHMIDT, |
| I. B. DAVENPORT, | JOHN DIDSBURY, |
| C. V. DU BOUCHET, | L. SAUSSINE, |
| CHARLES HOTZ, | GEORGE ROUSSEL, |
| THEODORE W. EVANS, | W. S. DANENPORT, |
| J. H. SPAULDING, | HENRY DIDSBURY. |

[1] "Dentine Plastique du Dr. Klein du Buda-Pesth. Vue de sa transformation dans la chambre pulpaire après trois années de séjour dans la bouche."

